# Local cellular immune response plays a key role in protecting chickens against hepatitis-hydropericardium syndrome (HHS) by vaccination with a recombinant fowl adenovirus (FAdV) chimeric fiber protein

**DOI:** 10.3389/fimmu.2022.1026233

**Published:** 2022-10-28

**Authors:** Carlotta De Luca, Anna Schachner, Sarah Heidl, Michael Hess, Dieter Liebhart, Taniya Mitra

**Affiliations:** ^1^ Christian Doppler Laboratory for Innovative Poultry Vaccines (IPOV), University of Veterinary Medicine, Vienna, Austria; ^2^ Clinic for Poultry and Fish Medicine, Department for Farm Animals and Veterinary Public Health, University of Veterinary Medicine, Vienna, Austria

**Keywords:** fowl adenovirus, FAdV-4, chimeric fiber vaccine, humoral immunity, cellular immunity, hepatitis-hydropericardium syndrome

## Abstract

Fowl adenovirus (FAdV)-induced diseases hepatitis-hydropericardium syndrome (HHS) and inclusion body hepatitis (IBH) have been affecting the poultry industry with increasing severity in the last two decades. Recently, a subunit vaccine based on a chimeric fiber protein with epitopes from different fowl adenovirus serotypes (named crecFib-4/11) has been shown to confer simultaneous protection against both HHS and IBH. However, the underlying immune mechanisms in chickens are still enigmatic, especially because of frequently absent neutralizing response despite high levels of protection. In this study, we investigated the kinetics of the humoral and cellular immune responses in specific pathogen-free chickens after vaccination with crecFib-4/11 and/or challenge with a HHS-causing strain, on a systemic level, as well as locally in target and lymphoid organs. The humoral response was assessed via enzyme-linked immunosorbent assay (ELISA) and virus neutralization test in serum, while the cellular immune response was determined by phenotyping using flow cytometry. Although vaccination induced serum antibodies, as confirmed by ELISA, such antibodies exhibited no pre-challenge neutralizing activity against FAdV-4. Nevertheless, immunized birds experienced a significant B cell increase in the liver upon challenge, remaining high throughout the experiment. Furthermore, vaccination stimulated the proliferation of cytotoxic T lymphocytes, with earlier circulation in the blood compared to the challenge control and subsequent increase in liver and spleen. Overall, these findings imply that protection of chickens from HHS after crecFib-4/11 vaccination relies on a prominent local immune response in the target organs, instead of circulating neutralizing antibodies.

## 1 Introduction

Fowl adenoviruses (FAdVs) are non-enveloped dsDNA viruses belonging to the family *Adenoviridae*, genus *Aviadenovirus* (1). They are classified into five species (*Fowl aviadenovirus A* to *Fowl aviadenovirus E*, FAdV-A to FAdV-E) based on genomic features, and 12 serotypes (FAdV-1 to -8a, -8b to -11) defined by cross-neutralization (2). Different FAdV species are responsible for three disease complexes affecting chickens: adenoviral gizzard erosion (AGE), caused by FAdV-A (serotype 1), hepatitis-hydropericardium syndrome (HHS), caused by FAdV-C (serotype 4), and inclusion body hepatitis (IBH), caused by FAdV-D (serotypes 2 and 11) and -E (serotypes 8a and 8b) with increasing importance in recent years (2, 3). Among these diseases, HHS and IBH share common pathogenesis and immunological features, as opposed to AGE (2, 4). Despite the worldwide occurrence of FAdVs and their growing economic impact, the lack of broadly protective and commercially accessible immunization strategies has led to extensive investigations on experimental vaccine antigens (3–7). In regards to subunit vaccines, the fiber protein, one of the major structural capsid components and surface antigens, has proven highly effective against HHS- and IBH-causing strains (8–15). Recently, the novel concept of chimeric fiber protein (crecFib) was formulated by designing a recombinant construct mimicking the full fiber structure incorporating epitopes from two distinct serotypes, in order to protect chickens from the diverse etiology of IBH (16). The concept was subsequently extended across FAdV species to obtain a singular vaccination antigen against IBH and HHS. The relevant chimeric construct containing epitopes from FAdV-4 and -11 fibers (named crecFib-4/11) provided coverage against both diseases (17). Even so, the immune mechanisms underlying such protection remain unclear. In fact, discrepancies between the potently neutralizing protective response against crecFib-8b/8a and an absent neutralizing activity of the crecFib-4/11 response, despite eliciting high protection, further complicate the matter (16, 17). It has been shown previously that live and fiber-based vaccines against FAdV-4 do not always lead to the development of neutralizing antibodies (nAbs), which suggests the involvement of alternative immune pathways (8, 17, 18). Recent studies have indicated a more substantial role of the cellular immune response not only in regards to live vaccines (18, 19), but for recombinant fiber antigens as well (9, 10, 13). Despite some preliminary data, investigations of the cellular immunity in FAdV subunit vaccines are, however, limited to peripheral blood mononuclear cells (PBMCs), and only to a few cell populations. Consequently, the local response within target and/or lymphoid organs is still entirely unknown. For this reason, we investigated the kinetics of a comprehensive panel of major immune cell populations in specific pathogen-free (SPF) chickens vaccinated with the novel crecFib-4/11, with or without subsequent FAdV-4 challenge. The investigation was performed on both circulating cells in blood, and locally in primary (thymus and bursa of Fabricius) and secondary (spleen) lymphoid organs, which are also infected by the virus, with the further addition of one of the major target organs of FAdV-4, the liver.

## 2 Materials and methods

### 2.1 Chimeric fiber vaccine and virus preparation

A chimeric fiber protein retaining epitopes from FAdV-4 and FAdV-11, named crecFib-4/11, was designed and expressed as previously described (16, 17). Briefly, the open reading frames of fibers from FAdV-4 (fib-2) (reference strain KR5, GenBank accession no. HE608152) and FAdV-11 (field isolate 13/14796) were divided into an amino- and a carboxy-distal segment, and assembled via Gibson assembly cloning. The recombinant protein crecFib-4/11 was expressed in *Spodoptera frugiperda* Sf9 cells using baculovirus system, and purified via polyhistidine tag on affinity chromatography columns (His GraviTrap, GE Healthcare, Freiburg, Germany) as described by Schachner et al. (8).

The FAdV-4 field isolate AG234 (GenBank accession no. MK572849) was applied as challenge strain for the animal experiment after being 3-fold plaque purified and propagated on primary chicken-embryo liver cells (20). Viral titers were determined by endpoint titration (21).

### 2.2 Animal experiment

The protection study was described previously in detail (17). Briefly, 80 SPF layer-type chicks were hatched, individually tagged and divided into four groups (n = 20) designed as: vaccinated only, vaccinated+challenged (vaccinated with crecFib-4/11 and challenged with FAdV-4), challenge control, and negative control (**Table 1**). Each group was housed separately in isolator units (HM2500, Montair, The Netherlands). One-day-old chicks received a 0.5 ml intramuscular injection of the vaccine containing 50 µg of crecFib-4/11 homogenized in a 40% (wt/vol) antigen oil-based adjuvant phase in the *Musculus iliotibialis lateralis*, whereas challenge control birds were injected with phosphate buffered saline (PBS) mixed with adjuvant, and the negative control group was administered sterile PBS only. An analogous procedure was repeated for all groups at 7-day-old for a booster vaccination. The challenge was performed intramuscularly at 22 days of life (15 days post booster, dpb) for the vaccinated+challenged and challenge control groups with 200 µl of 10^7^ tissue culture infectious dose 50 (TCID_50_)/ml of FAdV-4 strain AG234. After challenge, the birds were monitored daily for clinical signs. At 3, 5, 7 and 14 days post challenge (dpc), up to five birds per group were euthanized and submitted to necropsy, during which data and samples were collected to assess several protection endpoints as detailed below. Animals that had to be euthanized due to severe clinical signs were examined and sampled immediately. All procedures were discussed and approved by the institutional ethics and welfare committee and the national authority according to §§26ff. of Animal Experiments Act, Tierversuchsgesetz 2012 – TVG 20212 (license number: BMBWF GZ 68.205/0116-V/3b/2019).

**Table 1:** Experimental groups of the protection study.

group	vaccination(1 + 7-day-old)	challenge(22-day-old)
vaccinated only	crecFib-4/11	-^1^
vaccinated+challenged	crecFib-4/11	FAdV-4
challenge control	adjuvant only	FAdV-4
negative control	–	–

^1^not applied.

### 2.3 Protection endpoints and antibody development

Investigations to assess the protective efficacy of the vaccine included the measurement of aspartate transaminase (AST) in blood, organ-body weight ratio and viral load quantification in target and lymphoid organs through qPCR. The antibody development was investigated in serum with ELISA plates coated with the vaccination antigen and via virus neutralization (VN) test. All the methods have been described previously in detail (17). For the purpose of the present study, the viral load in thymus was investigated in addition to the data already available.

### 2.4 Histopathology

Samples from liver, spleen, thymus and bursa of Fabricius of birds euthanized at 4-5 dpc were collected during necropsy from the vaccinated+challenged, challenge control and negative control groups. The tissues were fixed in 4% neutral buffered formalin before being dehydrated and embedded in paraffin. Tissue sections of 5 µm thickness were cut with a microtome (Microm HM 360, Microm Laborgeräte GmbH, Walldorf, Germany) and mounted on glass slides before undergoing hematoxylin-eosin staining. Examination was performed using the Olympus BX53 microscope and documented with an Olympus DP72 camera (Olympus Corporation, Tokyo, Japan).

### 2.5 Cellular response

Blood for flow cytometry (FCM) analyses on PBMCs was collected from five birds per group before challenge, at 21-day-old (14 dpb). At different time points after challenge (3, 5, 7 and 14 dpc), FCM analyses were performed from mononuclear cells from blood, liver, spleen, thymus and bursa of Fabricius from three birds per group.

#### 2.5.1 Blood collection and preparation

For separation of PBMCs, 2 ml of blood were collected from the wing vein of each bird in a syringe containing 10% heparin (Serva, Heidelberg, Germany) at 21-day-old, and 4 ml from the jugular vein into a 10% heparin tube for the post-challenge time points. The blood was mixed with an equal volume of cold PBS, pH 7.4 (ThermoFisher Scientific, Vienna, Austria) with 2% fetal bovine serum (FBS) (ThermoFisher Scientific). The prepared suspension was then slowly layered above a double volume of Histopaque^®^-1077 (Sigma-Aldrich, Vienna, Austria) for density gradient centrifugation performed at room temperature, 350 x g for 30 min, without brake. Afterwards, the cells from the interphase layer were collected and washed three times through centrifugation, 400 x g for 10 min at 4°C, with full brake. Finally, the pellet was dissolved in 1 to 5 ml of cold PBS + 2% FBS.

#### 2.5.2 Preparation of liver, spleen, thymus and bursa of Fabricius

Single cell suspensions from the four organs were obtained by mechanical dissection (22). Briefly, isolation of lymphocytes was performed by squeezing the liver with the end of the plunger of a 20 ml syringe, and for the other organs by tearing apart the tissue with the help of two forceps in petri dishes containing up to 30 ml cold PBS + 2% FBS. The cells were then separated from the remaining tissue through a 40 µm nylon cell strainer (BD Falcon, BD Bioscience, San Jose, CA, USA) in a 50 ml tube. Following that, centrifugation was performed at room temperature, 350 x g for 10 min. The pellet was resuspended in 5 ml cold PBS + 2% FBS and separated by density gradient before washing as described for PBMCs. Mononuclear cells were finally resuspended in 5-10 ml cold PBS + 2% FBS.

#### 2.5.3 FCM staining protocol

Mononuclear cells from the blood and the analyzed organs were examined for their viability and quantity using Nexcelom cellometer X2 fluorescent viability cell counter system (Nexcelom Bioscience, Manchester, UK). A concentration of 2 x 10^7^ live cells/ml of PBS + 2% FBS was adjusted before further processing. Two different sets of monoclonal antibodies were used for immunophenotyping of live B cells (CD45^+^Bu1^+^), monocytes/macrophages (CD45^+^Kul01^+^), CD4^+^ T cells (CD45^+^CD4^+^CD8α^-^), CD8α^+^ T cells (CD45^+^CD4^-^CD8α^+^), and CD8α^+^TCRδγ^+^ T cells (CD45^+^CD4^-^CD8α^+^TCRδγ^+^) from the isolated cells. The gating strategy is given as additional figure (**Supplementary File 1**). A uniform gating hierarchy was used throughout all sampling days. Detailed information on antibody combinations, their fluorescence labelling strategy, manufacturers and catalogue number is given in **Supplementary File 2**. The final concentration of every antibody was determined by titration. The respective isotype controls and live cells without antibody staining were included for every group.

For staining of mononuclear cells, 25 µl of 2 x 10^7^ live cells/ml were transferred into each well of 96-well microtiter plates (Sarstedt, Nümbrecht, Germany) together with the respective primary antibodies for incubation for 20 min at 4°C. Afterwards, cell pellets obtained by centrifugation with full brake at 4°C, 450 x g for 4 min, were washed two times with cold PBS + 2% FBS. For biotinylated antibodies, the secondary reagent Brilliant Violet 421™ Streptavidin (BioLegend, San Diego, CA, USA) was applied. Following another incubation step for 20 min at 4°C, further washing was performed. The cells were fixed with BD fixation buffer (BD Biosciences) according to the manufacturer’s protocol. Finally, the pellets were resuspended in 100 µl cold PBS + 2% FBS and kept at 4°C until FCM analysis the following day.

#### 2.5.4 FCM analysis

FCM assay was conducted on the stained cells with a FACSCanto II (BD Biosciences) flow cytometer equipped by FACSDiva Software version 9.0 (BD Biosciences). At least 40,000 lymphocytes per sample were recorded. Analysis of FCM raw data was performed by FlowJo_V10 software (BD Biosciences).

### 2.6 Statistical analyses

A preliminary analysis of the datasets was carried out using Shapiro-Wilk test together with a visual inspection of histograms and normal Q-Q plots to assess normal distribution. Unpaired Student’s *t*-test was applied to compare the mean values for each cell population of the different experimental groups to the negative control, in addition to the previously published pathology parameters (17). Mann-Whitney *U* test was used for the datasets that did not meet the normality assumptions. In each case, *p* values ≤ 0.05 were considered statistically significant. Statistical analyses were performed with the software package SPSS Version 27 (IBM SPSS Statistics; IBM Corp., Armonk, New York, USA).

## 3 Results

### 3.1 Protection endpoints and antibody development

**Figure 1:**
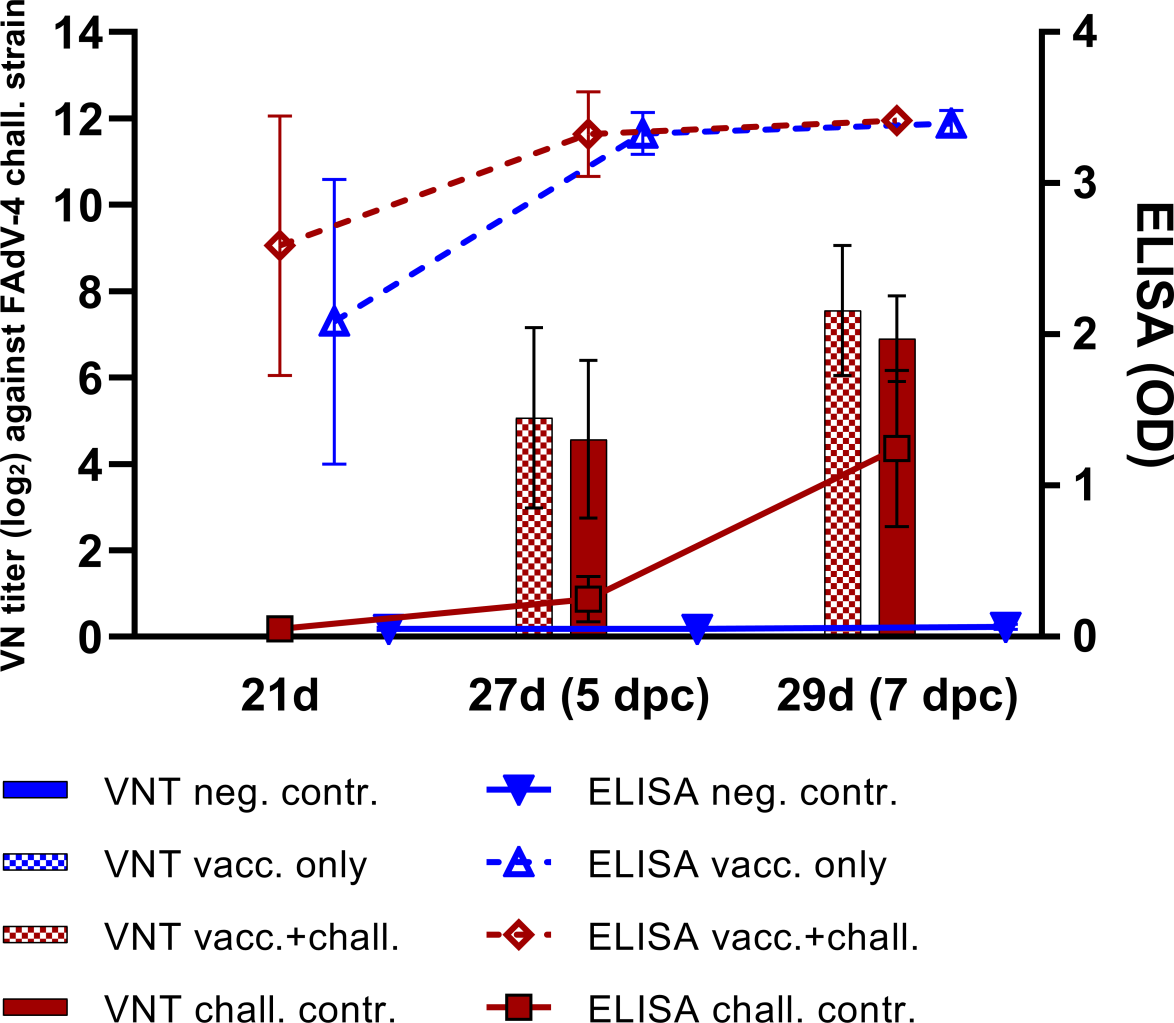
Antibody development following crecFib-4/11 vaccination in each experimental group of the animal study. The lines represent mean values of antibodies measured on ELISA plates coated with crecFib-4/11, whereas the bars indicate VN titers against the FAdV-4 challenge strain. No neutralizing antibodies were detected in the vaccinated only and the negative control groups at any time point. Reprinted from: Vaccination with a fowl adenovirus chimeric fiber protein (crecFib-4/11) simultaneously protects chickens against hepatitis-hydropericardium syndrome (HHS) and inclusion body hepatitis (IBH), Vaccine (2022) 40, De Luca C, Schachner A, Heidl S, Hess M, pages no. 1837–45, Copyright © 2022 Elsevier B.V. or its licensors or contributors, with permission from Elsevier (17).

Vaccination with crecFib-4/11 prevented clinical signs as opposed to the challenge control, and significantly limited hepatomegaly, atrophy of the lymphoid organs, and pathological rise of AST caused by HHS. Details on the values of each parameter for every experimental group have been previously published (17). Vaccination also reduced the viral load in liver, spleen, and bursa of Fabricius compared to the challenge control. However, no significant differences were observed in thymus between the vaccinated+challenged and the challenge control group at any time point. Mean viral copies/reaction values in thymus were the highest between 3 dpc (2.91 ± 1.98 log_10_ in vacc.+chall. vs. 2.09 ± 1.28 log_10_ in chall. contr.) and 5 dpc (2.05 ± 2.38 log_10_ vs. 3.33 ± 1.36 log_10_), before decreasing at 7 dpc (0.87 ± 1.95 log_10_ vs. 0.45 ± 0.63 log_10_). No virus was detected in thymus at 14 dpc for any of the investigated groups. As reported in our previous study, the vaccine induced a high and uniform antibody response measured on crecFib-4/11 ELISA before challenge, with the vaccinated groups plateauing at the highest measurable OD values by 25 days of age (corresponding to 3 dpc), independently from the challenge (17). Nevertheless, nAbs against FAdV-4 were only detectable after challenge in both challenged groups. The antibody development is summarized in **Figure 1**.

### 3.2 Histopathology

**Figure 2:**
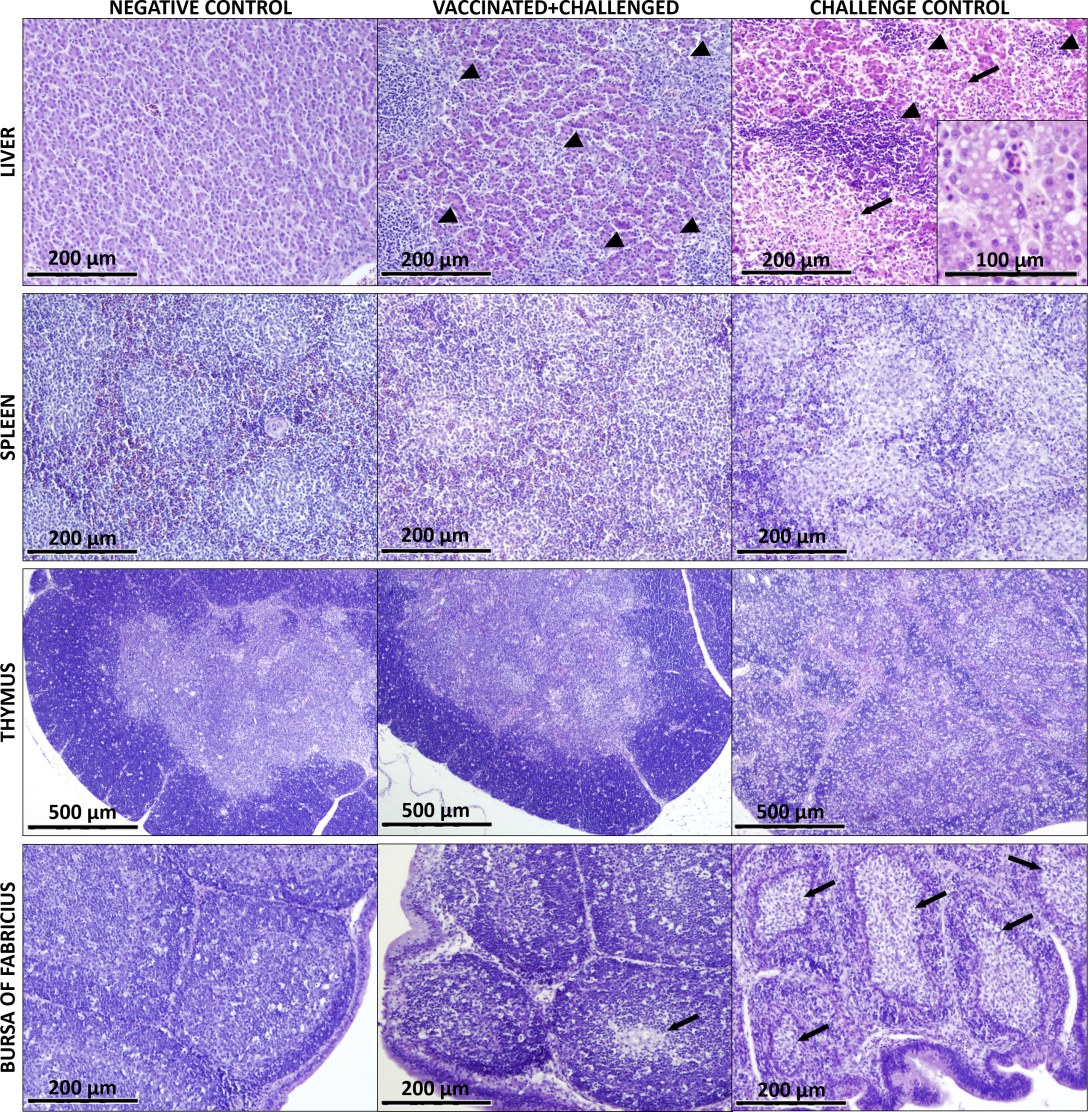
Main histological lesions observed in the analyzed organs of birds from negative control, vaccinated+challenged, and challenge control group. The liver of vaccinated+challenged group presents lymphocytic infiltration (marked by arrow heads) and small areas of degeneration (arrows), which can be seen more severe in the challenge control. Vacuolization of the hepatocytes is shown in a magnified field for the challenge control. Lymphocytic depletion is visible in the spleen and thymus of the challenge control group. The bursa of Fabricius of the vaccinated+challenged group shows mild lymphocyte depletion and few necrotic areas (arrows) in the medulla of the follicles, whereas the same lesions are much more pronounced and widespread in the challenge control.

The histological lesions highlighting differences between experimental groups are exemplarily shown in **Figure 2**. At 4-5 dpc, birds from the challenge control group presented extensive and severe microscopic lesions in the liver such as lymphocytic infiltration, necrotic areas and degeneration of hepatocytes with vacuolization. Furthermore, lymphocytic depletion was observed in spleen, thymus and bursa of Fabricius of challenge control birds, together with necrotic areas in the follicles of the bursa. Histological lesions were also observed in livers of vaccinated+challenged birds, although substantially reduced compared to only-challenged birds. In samples from vaccinated+challenged birds, lymphocytic depletion was not recorded in the spleen and thymus but it was sporadically noticed in their bursa of Fabricius. No lesions were observed in the organs of negative control birds.

### 3.3 Cellular immune response

#### 3.3.1 B cells

**Figure 3:**
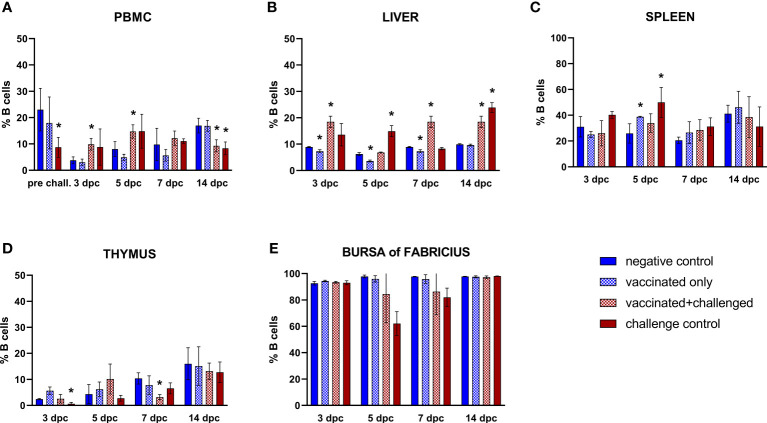
Relative number of B cells within total CD45^+^ cells measured via FCM for each experimental group throughout the study in PBMC **(A)**, liver **(B)**, spleen **(C)**, thymus **(D)**, and bursa of Fabricius **(E)**. Data for the two vaccinated groups (vaccinated only and vaccinated+challenged) are merged to determine values in PBMCs pre-challenge. Asterisks represent significant difference compared to the negative control at each time point (*p* ≤ 0.05).

In the PBMCs, B cell frequencies were significantly decreased before challenge compared to basal levels (always defined by the negative control) in the challenge control, which at this point comprised birds injected with adjuvant only (**Figure 3A**). After challenge, the priming effect of the vaccination was noticed at 3 and 5 dpc, with a significant rise of circulating B lymphocyte frequencies in the vaccinated+challenged group, before a drop was observed for both challenged groups at 14 dpc. The same priming effect of the vaccine was recorded in the liver, with a significant increase of B cells at 3, 7 and 14 dpc in the vaccinated+challenged group, whereas challenge control birds experienced a rise only at 5 and 14 dpc (**Figure 3B**). In the same organ, there was a significant decrease of hepatic B lymphocytes in the vaccinated only group from 3 to 7 dpc (corresponding to 18 to 22 dpb). The frequencies of splenic B cells significantly rose at 5 dpc in vaccinated only and challenge control birds, whereas a significant decrease was noticed in thymus at 3 dpc for the challenge control, and at 7 dpc in the vaccinated+challenged birds (**Figures 3C, D**). No relevant changes were recorded in the bursa of Fabricius (**Figure 3E**)

#### 3.3.2 Monocytes/macrophages

**Figure 4:**
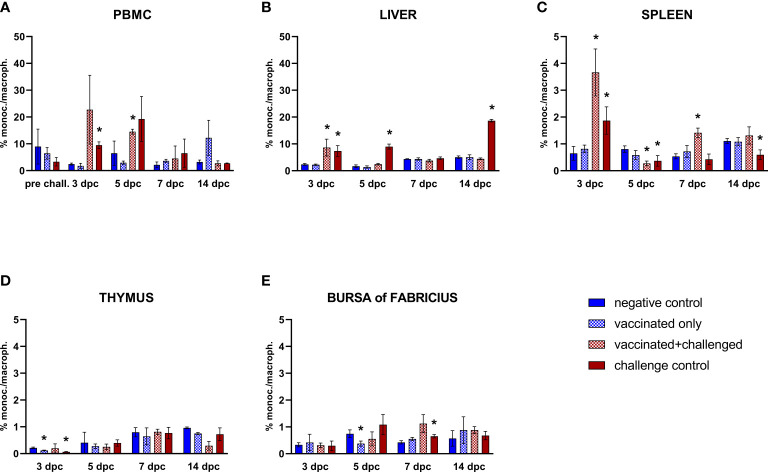
Relative number of monocytes/macrophages within total CD45^+^ cells measured *via* FCM for each experimental group throughout the study in PBMC **(A)**, liver **(B)**, spleen **(C)**, thymus **(D)**, and bursa of Fabricius **(E)**. Data for the two vaccinated groups (vaccinated only and vaccinated+challenged) are merged to determine values in PBMCs pre-challenge. Asterisks represent significant difference compared to the negative control at each time point (*p* ≤ 0.05).

The only significant variations in the frequencies of circulating monocytes/macrophages were limited to an increase in the challenge control and the vaccinated+challenged group at 3 and 5 dpc, respectively (**Figure 4A**). Subsequently, monocytes/macrophages frequencies consistently rose in the liver of challenge control birds at 3, 5 and 14 dpc, whereas vaccinated+challenged birds only experienced an increase in the early phase of infection (3 dpc) (**Figure 4B**). On the other hand, splenic monocytes/macrophages frequencies rose at 3 dpc and dropped below basal levels immediately after, at 5 dpc, in both challenged groups, before increasing once again in the vaccinated+challenged group (7 dpc), whereas they were still found significantly low in the challenge control at 14 dpc (**Figure 4C**). The vaccinated only and challenge control groups showed a significant decrease of monocytes/macrophages frequencies in thymus at 3 dpc compared to the negative control, and a drop was also observed in the bursa of Fabricius for vaccinated only birds (5 dpc, corresponding to 20 dpb), whereas challenge control birds showed a rise (7 dpc) (**Figures 4D, E**).

#### 3.3.3 CD4^+^ T cells

**Figure 5:**
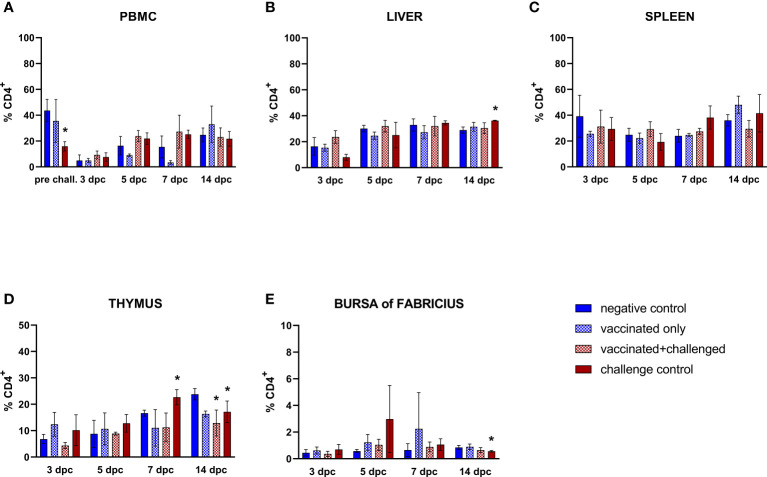
Relative number of CD4^+^ T lymphocytes within total CD45^+^ cells measured *via* FCM for each experimental group throughout the study in PBMC **(A)**, liver **(B)**, spleen **(C)**, thymus **(D)**, and bursa of Fabricius **(E)**. Data for the two vaccinated groups (vaccinated only and vaccinated+challenged) are merged to determine values in PBMCs pre-challenge. Asterisks represent significant difference compared to the negative control at each time point (*p* ≤ 0.05).

Similarly to the trend observed in the B cells, circulating CD4^+^ T lymphocyte frequencies of challenge control birds were significantly decreased compared to basal levels in PBMCs before challenge (**Figure 5A**). Aside from that, helper T cells (CD45^+^CD4^+^CD8α^-^) were only sporadically affected throughout the study. A late increase (14 dpc) was noticed in the frequencies of hepatic CD4^+^ T cells in the challenge control (**Figure 5B**). No changes were observed in the spleen (**Figure 5C**). Thymus and bursa of Fabricius presented a drop of this cell population in the challenge control at 14 dpc, after an earlier rise in the thymic population at 7 dpc (**Figures 5D, E**). A late significant decrease of CD4^+^ T cell frequencies was also observed in the thymus of vaccinated+challenged birds (14 dpc).

#### 3.3.4 CD8α^+^ T cells

**Figure 6:**
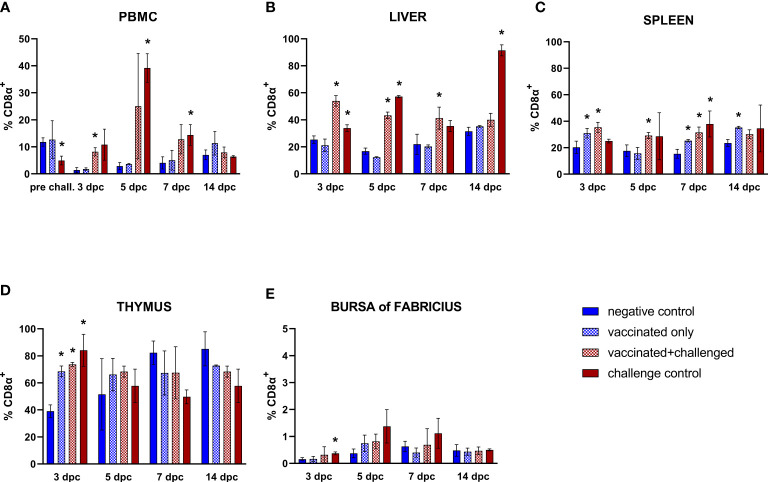
Relative number of CD8α^+^ T lymphocytes within total CD45^+^ cells measured *via* FCM for each experimental group throughout the study in PBMC **(A)**, liver **(B)**, spleen **(C)**, thymus **(D)**, and bursa of Fabricius **(E)**. Data for the two vaccinated groups (vaccinated only and vaccinated+challenged) are merged to determine values in PBMCs pre-challenge. Asterisks represent significant difference compared to the negative control at each time point (*p* ≤ 0.05).

Frequencies of circulating CD8α^+^ T cells suffered from a pre-challenge decrease in the challenge control (15 dpb) (**Figure 6A**). After challenge, the priming effect of vaccination was strongly recorded in PBMC CD8α^+^ T lymphocytes, whose frequency rose significantly in the vaccinated+challenged group at 3 dpc, before immediately returning to basal levels, whereas the challenge control responded with an increase that came later and lasted longer (5-7 dpc). In the liver, this trend was reflected with an early and prolonged rise of CD8α^+^ T lymphocytes of both challenged groups, although the challenge bird control still showed significantly higher hepatic cytotoxic T cells (CD45^+^CD4^-^CD8α^+^) at 14 dpc (**Figure 6B**). Both the vaccination and the challenge showed the tendency to stimulate CD8α^+^ T cells in the spleen, with a significant rise in the vaccinated+challenged group through the whole first week after challenge, and in the vaccinated only group at 3, 7, and as late as 14 dpc, whereas the challenge control group only showed an increase of splenic CD8α^+^ T lymphocytes at 7 dpc (**Figure 6C**). These rises were accompanied by an increase of this lymphocyte population frequency in the thymus of birds from all the three groups early after infection (3 dpc), which was also observed in the bursa of Fabricius of the challenge control birds (**Figures 6D, E**).

#### 3.3.5 CD8α^+^TCRγδ^+^ T cells

**Figure 7:**
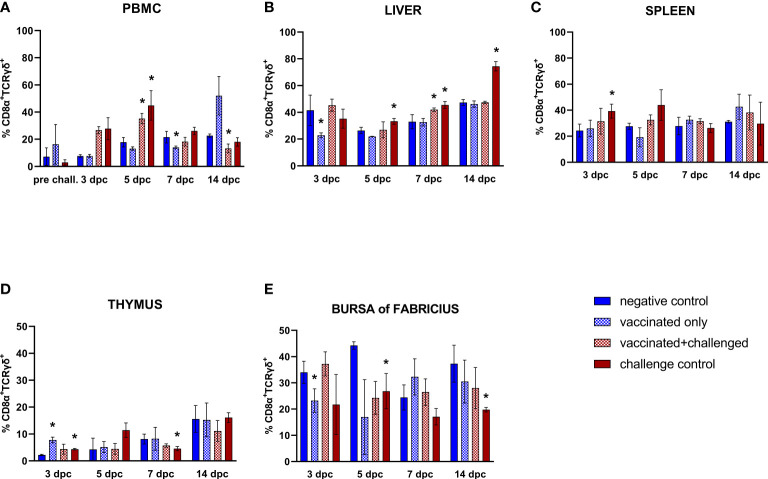
Relative number of CD8α^+^TCRγδ^+^ T lymphocytes within total CD8α^+^ T lymphocytes measured via FCM for each experimental group throughout the study in PBMC **(A)**, liver **(B)**, spleen **(C)**, thymus **(D)**, and bursa of Fabricius **(E)**. Data for the two vaccinated groups (vaccinated only and vaccinated+challenged) are merged to determine values in PBMCs pre-challenge. Asterisks represent significant difference compared to the negative control at each time point (*p* ≤ 0.05).

A significant increase of CD8α^+^TCRγδ^+^ T cell frequencies was observed in the PBMCs of challenged birds at 5 dpc, followed by a late drop at 14 dpc in the vaccinated+challenged group (**Figure 7A**). A decrease was also observed in the vaccinated only group in PBMCs at 7 dpc (corresponding to 22 dpb) and, before, in the liver at 3 dpc (corresponding to 18 dpb). Hepatic CD8α^+^TCRγδ^+^ T lymphocyte frequencies were significantly elevated in the challenge control from 5 to 14 dpc, whereas such increase was only observed at 7 dpc for the vaccinated+challenged group (**Figure 7B**). The challenge control was the only group showing increased CD8α^+^TCRγδ^+^ T cells in the spleen (3 dpc) (**Figure 7C**). A proliferation of these cells was noticed in the thymus of vaccinated only and challenge control birds at 3 dpc (corresponding to 18 dpb), before a drop was recorded at 7 dpc in the challenge control (**Figure 7D**). A decrease of CD8α^+^TCRγδ^+^ T cell frequencies in the bursa of Fabricius was observed in the vaccinated only group at 3 dpc, and in the challenge control at 5 and 14 dpc (**Figure 7E**).

## 4 Discussion

FAdV-related diseases such as HHS and IBH have proven to be particularly vexing to the poultry industry in the last two decades (3, 4). Recently, a novel concept based on recombinant chimeric fiber proteins, merging epitopes from different FAdV serotypes, was able to achieve broad coverage against both HHS and IBH, with a new construct retaining epitopes from FAdV-4 and -11 (crecFib-4/11) (17). However, the immune pathways associated with said protection are not fully elucidated. Whereas immunization from FAdV-8a and -8b is usually linked to a potent humoral response associated to the development of serotype-specific nAbs (9, 13, 16, 23–25), neutralizing activity seems to be dispensable when it comes to vaccines against FAdV-4 (8, 18, 19). This led to speculations over the potentially crucial role of the cellular immune response against HHS infections. Despite the numerous studies describing cytokines development over the course of FAdV-4 infection, which confirmed an important involvement of the cellular immune response (26), there is very little information available over the kinetics of the immune cell populations themselves. In particular, to date, no studies have focused on unravelling the development of both systemic and local cellular response related to FAdV-4 fiber vaccines before and after challenge. Therefore, in the present work, we investigated the kinetics of the major immune cell populations after vaccination with crecFib-4/11 and/or HHS challenge, in not only PBMCs, but also taking into account target and lymphoid organs of the birds over the course of the disease.

Vaccination with crecFib-4/11 resulted into high levels of systemic ELISA-measured antibodies against the vaccine antigen, but no nAbs against FAdV-4. Nevertheless, immunized birds challenged with virulent FAdV-4 showed a significant reduction of clinical signs and pathological lesions (17). This marks a substantial difference compared to fiber vaccines developed from FAdV-8a and -8b fibers, whose protection against IBH correlates with the presence of serotype-specific nAbs (9, 13, 16). High levels of antibodies after FAdV-8a fiber vaccination have been linked to a significant increase of B cells in blood before challenge (13), whereas in the present study, the increase of circulating B cells compared to basal levels (defined by the negative control) was noted in vaccinated birds only after challenge. A prominent rise of B lymphocytes was also observed in the liver of crecFib-4/11-vaccinated birds throughout the whole period after challenge, conforming with the hepatic lymphoid infiltration observed in histological analyses. The immediate increase of these cells in the blood of vaccinated+challenged birds, and subsequently in one of the major target organs, the liver, highlights the priming effect of vaccination. In fact, the kinetics of B lymphocytes in challenge control birds evolved differently, with an increase in the hepatic and splenic cell population after an initial depletion in the thymus, without a rise in circulating B cell frequencies. A decrease in thymus was also observed in the present study for immunized birds a week after challenge, possibly due to the proliferation of other immune cell populations in response to infection. Furthermore, a drop in the frequency of circulating B lymphocytes was noted in both challenged groups two weeks after challenge, independently from previous vaccination, possibly due to the migration of these cells in the liver. Notably, even with histological investigations highlighting the lymphocyte depletion in challenged groups, no significant changes were noticed in B cell frequencies of the bursa of Fabricius for any experimental group, confirming an earlier study published by Schonewille et al. (19) in birds infected with the same challenge strain. This may be because the relative count of B lymphocytes, which in the bursa of Fabricius constitute at least 98% of the whole lymphocyte population (27), did not suffer significant changes despite the overall depletion.

Monocytes/macrophages were also reduced in thymus of challenge control birds in the early phase of infection, while they rose in blood, liver and spleen of both vaccinated+challenged and challenge control birds. This marks a difference compared to FAdV-8a fiber vaccine, which did not elicit such rise in the blood of SPF broilers in response to an IBH infection, as opposed to the SPF layers utilized in the present work (13). In the challenge control, levels of hepatic monocytes/macrophages remained elevated longer than in the vaccinated+challenged group. At the same time, after an early increase, the infection caused a depletion of these antigen-presenting cells in the spleen, which was quickly resolved only in previously immunized birds.

No significant differences were observed in levels of CD4^+^ T lymphocytes in blood or spleen of vaccinated and/or challenged birds, contrary to previous studies, where both FAdV-4 (fib-2) and FAdV-8a fiber vaccines were shown to promote the proliferation of helper T cells in the blood of chickens (10, 13). Apart from the differences in the vaccination antigens, this discrepancy may also be explained by divergences between the adjuvant used, the age and, in particular, the type of birds. However, the same challenge strain (AG234) caused a drop in splenic CD4^+^ T cells in unvaccinated chickens in a previous study (19). This may be ascribable to the inconsistencies in pathogenicity already observed between *in vivo* experiments utilizing said challenge strain, possibly due to underlying differences between the batches of SPF birds (8, 17). Nevertheless, the crecFib-4/11 vaccine was able to prevent a late drop of CD4^+^ T lymphocytes in the bursa of Fabricius, as observed in the challenge control, although a decrease in thymus was recorded in both challenged groups two weeks after infection, despite the absence of viral load in the organ at this time point. Atrophy and lymphocyte depletion were repeatedly reported in the thymus and bursa of Fabricius in relation to HHS infection as a result of the immunosuppressive nature of the virus (17, 19, 28–33). However, in the present study, histological analyses showed that the crecFib-4/11 vaccine was able to limit HHS-caused lymphocyte depletion in immunized birds, a feature shared among successful experimental vaccines against FAdV-4 (19, 28, 33). Furthermore, cytotoxic CD8α^+^ T cell frequencies were never found significantly decreased compared to basal levels in any of the experimental groups after challenge. On the contrary, the inclusion of a vaccinated only group allowed us to detect the proliferation of CD8α^+^ T lymphocytes in thymus at 18 dpb, and in spleen as late as 29 dpb, which conforms to previous observations related to FAdV-E fiber vaccines inducing an increase of cytotoxic T lymphocytes, albeit in PBMCs (9, 13). In fact, the present work highlights the pivotal role of CD8α^+^ T cells in protecting chickens against HHS, thanks to the immediate rise observed in the thymus and liver of challenged birds, and the earlier increase of circulating and splenic cytotoxic T cells in vaccinated birds compared to the challenge control. On the other hand, frequencies of CD8α^+^TCRγδ^+^ lymphocyte subpopulation increased in the blood and liver of both challenged groups, although vaccinated+challenged birds did not show a previous proliferation in thymus and spleen, nor suffered a drop in primary lymphoid organs, as opposed to the challenge control. Prolonged exposure to an antigen can result in T cell exhaustion, which is characterized by the loss of T cell effector function (34). In fact, we demonstrated that FAdV-4 can persist up to two weeks after infection in the spleen (17). These findings may also relate to the downregulation of certain splenic cytokines, such as IL-18 and INF-γ, during the late phase of infection (35), confirming that FAdV-4 viruses reflecting different pathogenicity can persist in this organ even after the acute phase of infection, thus extending a condition of subclinical immunosuppression in the birds.

Interestingly, many of the analyzed cell populations were affected by the adjuvant with a significant decrease in the blood after booster vaccination (14 dpb) compared to basal levels, as observed in the challenge control group that, at this point, had been injected with adjuvant only. This may be due to the adjuvant-induced recruitment of these immune cells to the injection site (36). Furthermore, the process was possibly enhanced by the local inflammation at the site of injection, which was still detectable during necropsy in the inoculated birds. Sporadic drops in the frequencies of B cells, monocytes/macrophages and CD8α^+^TCRγδ^+^ T lymphocytes were also recorded in various organs of the vaccinated only group such as PBMCs, liver, thymus and bursa of Fabricius. Although the reason behind this phenomenon is not clear, we hypothesize that the vaccine/adjuvant combination tailored the immune responsiveness towards cytotoxic CD8α^+^ T cells. Contrary to FAdV-8a-induced IBH in broilers, HHS infection in layers did not cause a prolonged decrease of monocytes/macrophages, nor a significant rise of CD4^+^ T lymphocytes in the blood over the course of the infection (13). It is known, in fact, that differences in the genetic background of the birds greatly influence their susceptibility to FAdVs (37). However, these contrasts may also be linked to FAdV-specific immune pathways, stressing the importance of broad-protective vaccines such as crecFib-4/11.

Overall, the observed priming and proliferation of immune cells after crecFib-4/11 immunization highlight the need for further investigations over the role of vaccine-induced antigen-specific antibodies. In fact, the importance of non-neutralizing antibodies (non-nAbs) has already been proven for vaccines against avian influenza virus (38–41). Therefore, it is possible that the non-nAbs induced by fiber vaccination may constitute a bridge between humoral and cellular immune response through mechanisms such as antibody-dependent cellular cytotoxicity (ADCC) and antibody-dependent cellular phagocytosis (ADCP), reflected by proliferation of macrophages and cytotoxic T cells in the present study.

In conclusion, despite the lack of circulating nAbs against FAdV-4, protection against HHS provided by crecFib-4/11 was characterized by a prominent and prolonged increase of B lymphocytes in one of the major target organs, the liver. Furthermore, the vaccine-induced increase of cytotoxic T lymphocytes, both circulating and in target organs, such as liver, spleen and thymus, highlights for the first time the pivotal role of local, cell-mediated immune response involved in protection against HHS after priming by FAdV fiber vaccine.

## Data availability statement

The original contributions presented in the study are included in the article/**Supplementary Material**. Further inquiries can be directed to the corresponding author.

## Ethics statement

The animal study was reviewed and approved by institutional ethics and welfare committee and the national authority according to §§26ff. of Animal Experiments Act, Tierversuchsgesetz 2012 – TVG 20212 (license number: BMBWF GZ 68.205/0116-V/3b/2019).

## Author contributions

TM, MH, DL, CDL, and AS conceived and designed the work. CDL and AS performed the animal trial. CDL, TM, and SH performed the laboratory analysis. CDL, TM, DL, AS, and MH interpreted the data. CDL drafted the manuscript. TM, DL, AS, and MH revised the manuscript critically for important intellectual content. All authors read and approved the final manuscript. All authors contributed to the article and approved the submitted version.

## Acknowledgments

The authors thank Dr. Erwin Mombarg from Vaxxinova^®^ International BV for providing the formulation of the vaccine and mock solutions used during the animal experiment. Furthermore, the authors wish to thank Sina Bagheri, Amin Mirzazadehghassab, Mohammad Deyaa Almasri, Valentin Frötscher, Attila Sandor and Vesna Stanisavljevic for assisting in the animal experiment.

## Conflict of interest

MH and AS declare the following financial interest that may be considered as potential competing interests: Patent ‘‘Fowl adenovirus subunit vaccine and production method thereof” pending to European Patent Office.

The remaining authors declare that the research was conducted in the absence of any commercial or financial relationships that could be construed as a potential conflict of interest.

The authors declare that this study received funding from the Christian Doppler Research Association, in cooperation with Vaxxinova GmbH, Münster, Germany (grant nr.: 189). Vaxxinova GmbH had the following involvement in the study: preparation of vaccine formulation and mock solution (adjuvant only).

## Publisher’s note

All claims expressed in this article are solely those of the authors and do not necessarily represent those of their affiliated organizations, or those of the publisher, the editors and the reviewers. Any product that may be evaluated in this article, or claim that may be made by its manufacturer, is not guaranteed or endorsed by the publisher.

## Abbreviations

AGE, adenoviral gizzard erosion; AST, aspartate transaminase; dpb, days post booster; dpc, days post challenge; ELISA, enzyme-linked immunosorbent assay; FAdV, fowl adenovirus; FBS, fetal bovine serum; FCM, flow cytometry; HHS, hepatitis-hydropericardium syndrome; IBH, inclusion body hepatitis; nAbs, neutralizing antibodies; non-nAbs, non neutralizing antibodies; PBMCs, peripheral blood mononuclear cells; PBS, phosphate buffered saline; SPF, specific pathogen-free; TCID_50_, 50% tissue culture infective dose; TCR, T cell receptor; VN, virus neutralization.
