# Supplementary material for: Local cellular immune response plays a key role in protecting chickens against hepatitis-hydropericardium syndrome (HHS) by vaccination with a recombinant fowl adenovirus (FAdV) chimeric fiber protein
Source: Front Immunol. 2022 Oct 28;13:1026233. doi: 10.3389/fimmu.2022.1026233 (PMC9650998; doi:10.3389/fimmu.2022.1026233)
Supplement: Supplementary file 1 [file Table_1.docx]

**Supplementary file 2.** Complete panels of antibody combinations utilized in the study and their fluorescence labelling strategy

| **antigen** | **clone (isotype)** | **fluorochrome/label** | **labelling strategy** | **source of primary monoclonal antibody** | **catalogue number** |
| --- | --- | --- | --- | --- | --- |
| **panel 1** | | | |  |  |
| CD45 | LT40 (IgM) | APC | direct conjugation | Southern-Biotech | 8270-11 |
| CD4 | CT4 (IgG1) | PE-cy7 | direct conjugation | Southern-Biotech | 8210-17 |
| CD8α | 3-298 (IgG2b) | PE | direct conjugation | Southern-Biotech | 8405-09 |
| TCR γδ | TCR1 (IgG1) | BIOTIN | biotin-streptavidin^a^ conjugation | Southern-Biotech | 8230-08 |
| live/dead staining | -^b^ | APC Cy7 | direct conjugation | BD Horizon | 565388 |
| **panel 2** | | | |  |  |
| CD45 | LT40 (IgM) | APC | direct conjugation | Southern-Biotech | 8270-11 |
| Bu1 | AV20 (IgG1) | PacBlu | direct conjugation | Southern-Biotech | 8395-26 |
| Kul01 | Kul01 (IgG1) | PE | direct conjugation | Southern-Biotech | 8420-09 |
| live/dead staining | - | APC Cy7 | direct conjugation | BD Horizon | 565388 |

^a^Brilliant Violet 421™ Streptavidin, BioLegend

^b^not applicable
