# Supplementary material for: Local cellular immune response plays a key role in protecting chickens against hepatitis-hydropericardium syndrome (HHS) by vaccination with a recombinant fowl adenovirus (FAdV) chimeric fiber protein
Source: Front Immunol. 2022 Oct 28;13:1026233. doi: 10.3389/fimmu.2022.1026233 (PMC9650998; doi:10.3389/fimmu.2022.1026233)
Supplement: Supplementary file 2 [file Presentation_1.pptx]

## Slide 1
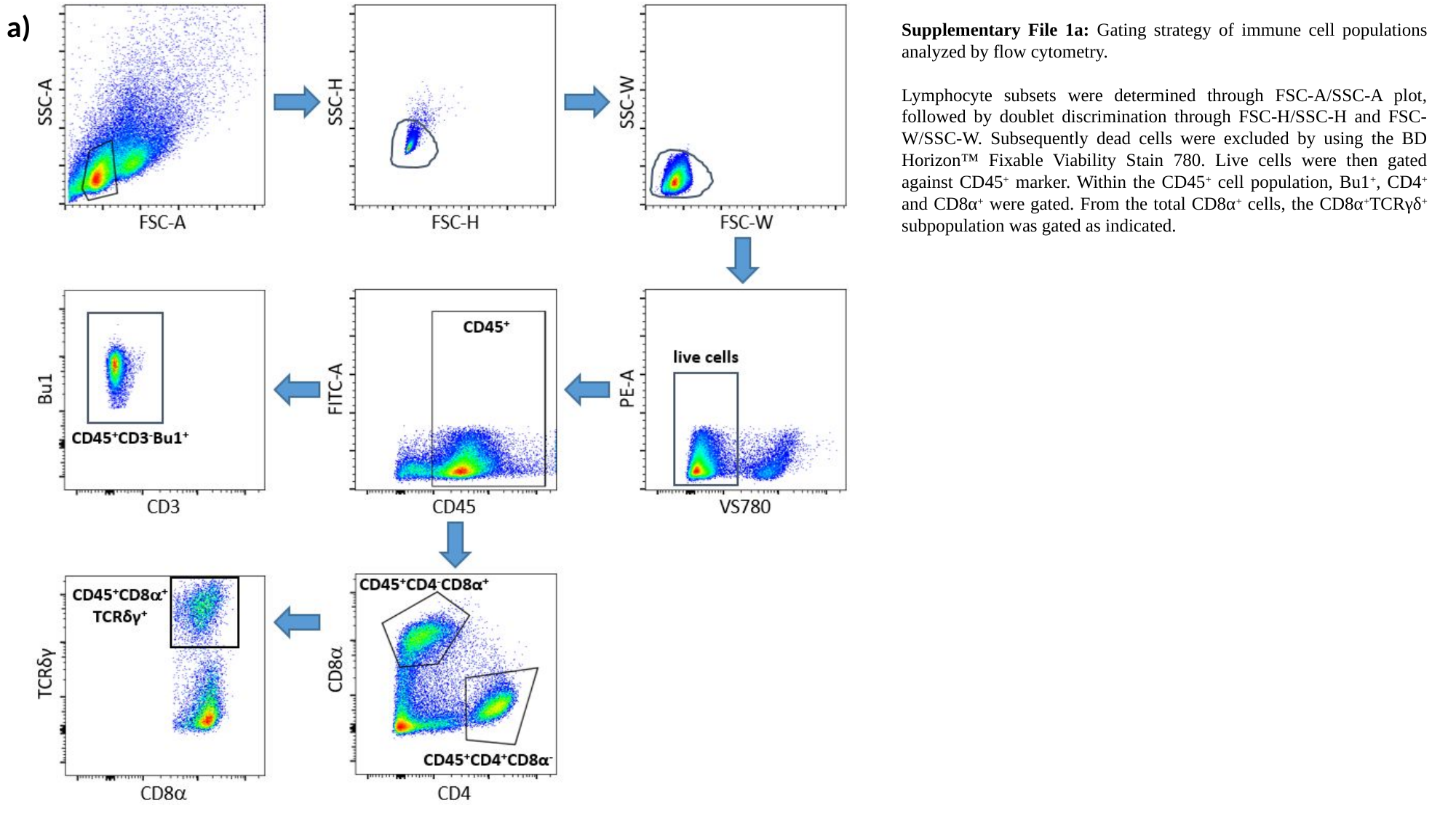

a)
Supplementary File 1a: Gating strategy of immune cell populations analyzed by flow cytometry.
Lymphocyte subsets were determined through FSC-A/SSC-A plot, followed by doublet discrimination through FSC-H/SSC-H and FSC-W/SSC-W. Subsequently dead cells were excluded by using the BD Horizon™ Fixable Viability Stain 780. Live cells were then gated against CD45+ marker. Within the CD45+ cell population, Bu1+, CD4+ and CD8α+ were gated. From the total CD8α+ cells, the CD8α+TCRγδ+ subpopulation was gated as indicated.

## Slide 2
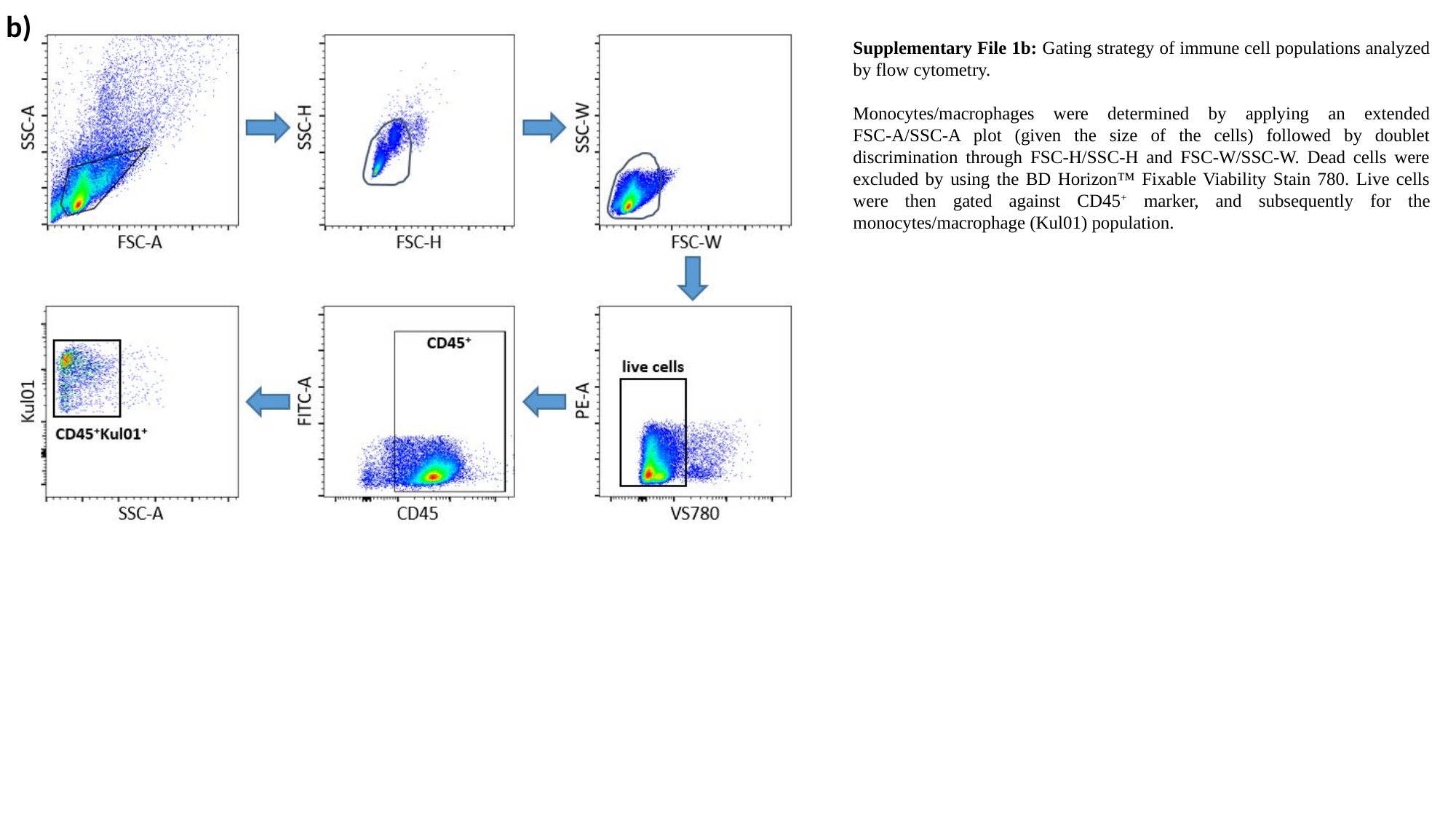

b)
Supplementary File 1b: Gating strategy of immune cell populations analyzed by flow cytometry.
Monocytes/macrophages were determined by applying an extended FSC-A/SSC-A plot (given the size of the cells) followed by doublet discrimination through FSC-H/SSC-H and FSC-W/SSC-W. Dead cells were excluded by using the BD Horizon™ Fixable Viability Stain 780. Live cells were then gated against CD45+ marker, and subsequently for the monocytes/macrophage (Kul01) population.
